# Age-dependent carry-over effects in a long-distance migratory bird

**DOI:** 10.1038/s41598-019-47374-3

**Published:** 2019-08-19

**Authors:** Cosme López Calderón, Javier Balbontín Arenas, Keith A. Hobson, Anders Pape Møller

**Affiliations:** 1 Department of Zoology, Faculty of Biology, Green Building, Avenue Reina Mercedes, E-41012 Seville, Spain; 2 Department of Biology, University of Western Ontario, London, Ontario Canada; 3 Ecologie Systématique Evolution, Université Paris-Sud, CNRS, AgroParisTech, Université Paris-Saclay, F-91405 Orsay Cedex, France

**Keywords:** Animal migration, Animal migration

## Abstract

Migratory birds usually respond to climate change by modifying breeding and/or wintering areas, as well as by reproducing earlier. In addition, changes in winter habitat use or breeding phenology could have important carry-over effects on subsequent breeding success. Here, we studied age- and sex-dependent carry-over effects from wintering to the breeding stage of a small aerial insectivorous long-distance migratory bird, the barn swallows (*Hirundo rustica*) breeding in Denmark during 1984–2013. First, we used stable isotope analyses combined with ringing recoveries to identify wintering areas. Second, we found that environmental conditions as inferred by Normalized Difference Vegetation Index (NDVI) have improved at the wintering grounds. Third, we used confirmatory path analysis to quantify the indirect effect of winter conditions on subsequent breeding success. Males delayed onset of breeding and raised fewer fledglings in the first brood when ecological conditions during the previous winter improved. This response was age dependent, since yearlings did not respond to this environmental cue but the response was increasingly stronger as males aged. Females showed a similar response to winter conditions, although not statistically significant. These results highlight the importance of studying carry-over effects within the context of climate change, especially in relation to age of individuals.

## Introduction

Many species of birds, especially those breeding at high latitudes, have adapted to perform regular annual movements between breeding and non-breeding areas, often involving thousands of kilometers^1^. Migratory behavior is important because, in an ecological context, the previous history and experience of a given individual may explain its performance in the current situation (i.e. “carry-over effect”)^2^. Hence, in migratory birds, what occurs during winter and the migratory journey could have consequences for subsequent reproductive success back at the breeding areas, which in turn, may result from a response to previous environmental conditions^3,4^. Furthermore, carry-over effects from the winter to the breeding stage may change according to age and sex classes^5–11^. Environmental conditions experienced in winter should first affect physical condition during winter, then timing of departure from wintering sites and ultimately timing of arrival to breeding areas^12,13^. In addition, arrival time to breeding areas usually determines the start of breeding, and this in turn influences the number of offspring produced^14–16^. Because all these variables flow sequentially in a time series fashion, Structural Equation Models (hereafter: SEM) provide a powerful and elegant approach to investigate carry-over effects in migratory birds^17–21^.

Differential migration among age and sex groups may arise because of the different role of each sex in reproduction, timing of other events during the annual cycle (e.g. moult) or factors related to body size and behavioral dominance^1^. For instance, males usually arrive at breeding areas earlier than females, a consequence of sexual selection through female choice^1^. Because females usually select partners that are established in the most suitable nesting habitats, competition for earlier arrival to breeding areas is higher among males, and they can develop different strategies for this purpose^22–24^. For example, males can stay closer to breeding areas during winter, advance departure date from winter areas, travel more quickly during spring migration or compete more aggressively for food^1^. In addition, young adults breeding for the first time usually arrive later to the breeding areas and have lower breeding success than more experienced adults. Age-related migratory behavior may differ for several reasons. For example, dominant (e.g. older) individuals could displace subordinates (e.g. younger) from better winter or stopover sites. In such cases, older individuals require less time to acquire fuel reserves or to complete moult, allowing older individuals to depart earlier from the wintering areas or to speed up the return journey (the so-called dominance hypothesis)^1,25^. On the other hand, individuals should gain competence through experience as they age, allowing them to perform the migratory journey better or to respond more eficiently against environmental conditions found in the wintering areas (the so-called constraint hypothesis)^26,27^. There is evidence supporting both hypotheses that are not mutually exclusive^1,27^. Alternatively, a third explanation for variation in migratory behavior among age classes is the disappearance from the population of individuals according to their quality (the so-called selection hypothesis)^26,27^. For example, an improvement with age in migratory performance at the population level (e.g. earlier arrival to the breeding areas) could be explained by the disappearance from the population of later arriving individuals (than average) as they age (i.e. removal of poorer quality individuals in relation to migratory performance).

Many different climate variables (e.g. temperature and precipitation) are changing rapidly worldwide and are predicted to continue to change in future years^28^. The use of biotic indices are especially powerful to understand and predict the indirect effect of climate change on organisms; such is the case of the Normalized Differential Vegetation Index (hereafter: NDVI)^29,30^. NDVI integrates the effect of climate on vegetation and vegetation dynamics in turn are linked to animal distribution and performance. For example, NDVI is linked to abundance and diversity of insects^31–33^. Associations between NDVI and climatic variables shift throughout the world. For instance, in tropical latitudes lower temperature and higher rainfall are usually correlated with higher NDVI^34^. Therefore, lower temperature and higher rainfall may result in greener vegetation, higher abundance of insects^31,33,35,36^, and thus favorable conditions for aerial insectivores wintering in the tropics, whereas higher temperatures and lower precipitation may result in the opposite^5,7,37–39^.

The barn swallow (*Hirundo rustica*) is a small passerine (*c*. 20 g) that breeds semi-colonially and feeds on insects while flying^7,18^. This species is a long-distance migrant that usually breeds north of the Tropic of Cancer and winters south of this latitude throughout the world^40,41^. For example, the Danish breeding population covers >6000 km to reach Central and South Africa^40^. Barn swallows provide excellent longitudinal data because once recruited to the breeding population, individuals almost always return to the same colony to breed every year^5,8,23^. In addition, first reproduction occurs at the first year, and therefore, a high trapping effort each year allows the estimatation of age with accuracy in this species^5,6,8^.

In this study, first we used both stable isotope analyses and ringing recoveries to determine the winter areas used by our study populations of barn swallows breeding in Denmark. Second, we tested if winter conditions based on satellite-derived NDVI have changed during the last three decades corresponding to the data available on our barn swallow study populations (i.e. 1984–2013). Third, we focused on how environmental conditions experienced in wintering areas, based on NDVI, affected subsequent breeding date and reproductive success measured as clutch size and number of fledglings in the first brood. We were interested to determine how the response to external factors affected these life history traits during the lifetime of known-aged individuals over the 30-year period (see Table 1 for sample sizes).

**Table 1:** Sample size across age class for each sex.

Age (years)	Females	Males
1	1414	1273
2	349	403
3	144	175
4	44	64
5	23	23
6	6	8
7	1	3
Total:	1981	1949

All our dependent variables of interest had the same sample size.

To explain age-related difference in response to conditions found at winter areas and subsequent carry-over effects during the breeding period, we set *a priori* predictions from established hypotheses as described above. Many previous studies have emphasized that earlier arrival to a breeding colony is associated with earlier breeding date and higher reproductive success of migratory birds^14,15,23,24^. Individuals may thus depart earlier from the wintering grounds in order to arrive earlier at the breeding areas^12,42,43^. To date it is well known that timing of migration depends on external environmental cues such as photoperiod^44^, weather conditions^1,45^ or winter habitat quality^12,13^. For instance, Coppack and collaborators^44^ experimentally analyzed the onset of migratory activity in the European pied flycatcher (*Ficedula hypoleuca*). These authors simulated different wintering latitudes by exposing birds to different photoperiods, and they found that a moderate increase in wintering latitude from 10° to 20°N resulted in a pronounced advancement in the termination of molt, initiation of migratory activity and gonadal growth. As another example, American redstarts (*Setophaga ruticilla*) wintering in Jamaica start spring migration as soon as they acquire the adequate body condition to withstand the migratory journey, which in turn depends on the quality of the habitat chosen for wintering and also on internal factors such as behavioral dominance among age and sex classes^12,13^. If we assume that individuals depart from their wintering areas as soon as possible after acquiring the appropriate body condition to start the migratory journey, then under the competition hypothesis we should expect a smaller age-related difference in breeding date in good compared to poor years. This outcome is expected because during good years competition is relaxed, and there should be less difference in breeding date amongst individuals of different ages. If it is the inexperience of young individuals that causes a delay in the onset of breeding, then environmental conditions experienced during winter should be less important, and we would expect similar age-related differences in breeding date with individuals breeding earlier as they age (i.e. constraint hypothesis).

## Results

### Wintering areas

**Figure 1:**
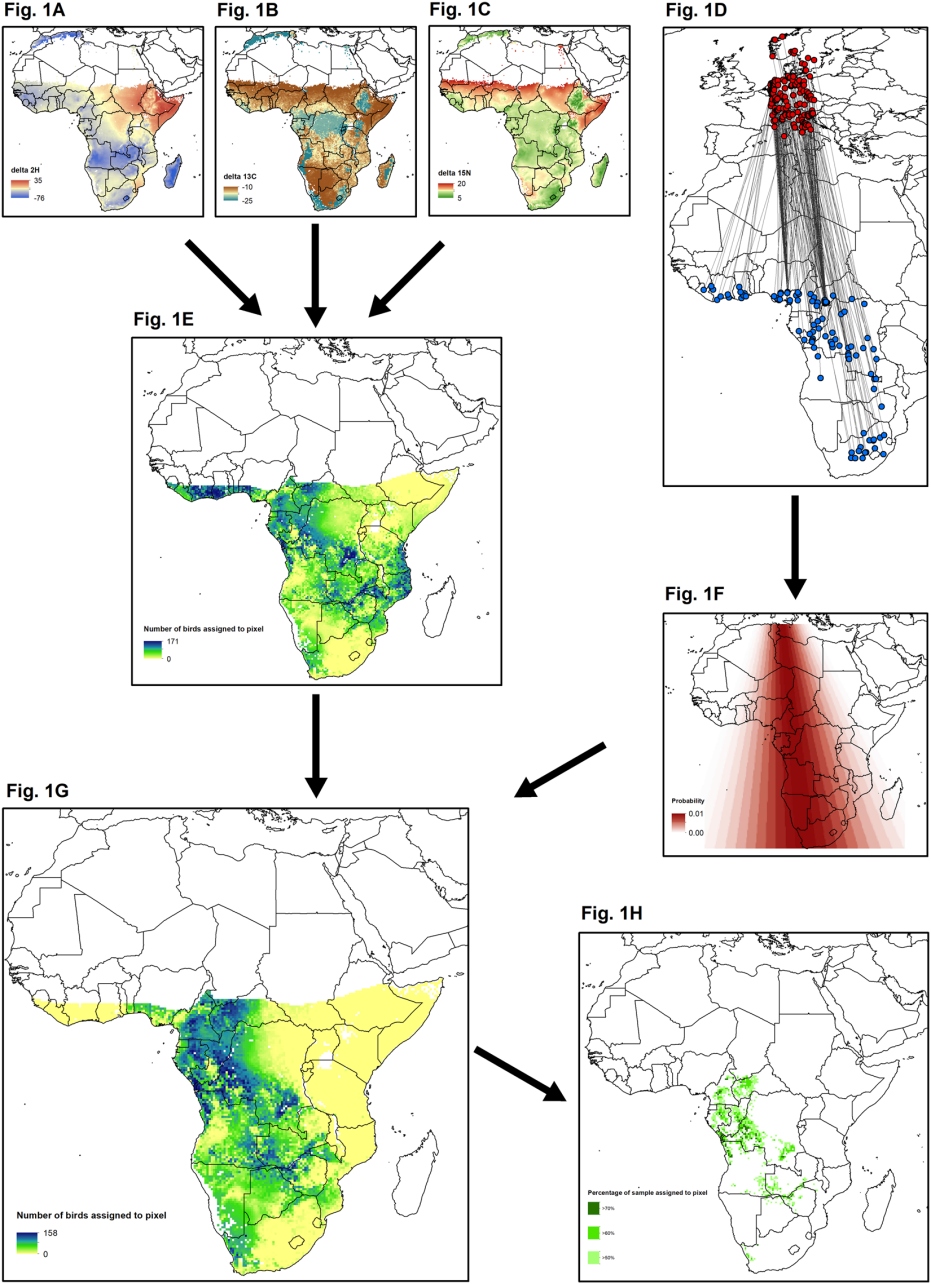
Summary of the full process developed to identify the wintering areas of our barn swallow populations breeding in Denmark. (**A**–**C**) Represent *δ*^2^H, *δ*^13^C and *δ*^15^N feather isoscapes^73^. (**D**) Represents breeding (red circles) and wintering (blue circles) locations for a given individual breeding between 5°E and 15°E longitude^60^. (**E**) Represents the population likely origin of our sample individuals based only on the multivariate assignment of *δ*^2^H, *δ*^13^C and *δ*^15^N. (**F**) Represents the prior surface obtained from fitting a von Mises probability density function to bearings connecting breeding and wintering locations. (**G**) Represents the likely origin population of our sample of individuals after incorporating the prior surface into the multivariate assignment of *δ*^2^H, *δ*^13^C and *δ*^15^N. (**H**) Represents the pixels that were assigned to be the likely origin for >50, >60 and 70% of our sample. These three spatial scales were then used to extract NDVI values. All maps were generated in ArcGis 10.2.2. (https://support.esri.com/es/products/desktop/arcgis-desktop/arcmap/10-2-2), but raster computations were conducted in *R*^65^.

All the processes we followed to combine ringing data with stable isotope values are summarized in Fig. 1. The prior surface derived from movement directions effectively narrowed the potential moult origin of our population of barn swallows. Specifically, Fig. 1E represents the potential origins based only on the multivariate assignment of *δ*^2^H, *δ*^13^C and *δ*^15^N, but after having incorporated the prior surface within the calculation of the spatially explicit probability density maps for each individual (*fx*), the potential origins for the population are narrowed from 10°E to 30°E (Fig. 1G,H). Wintering areas were largely spread through Cameroon, Central African Republic, Gabon, Congo, Democratic Republic of Congo, Angola, Zambia, Zimbabwe and some patches across Botswana and South Africa (Fig. 1H). Total surface of the wintering area was 1,370,090 km^2^, 492,257 km^2^ and 97,621 km^2^ for our different spatial scales (respectively pixels assigned to be the origin for >50, >60 and >70% of our sample). African biomes dominating the wintering areas were mainly equatorial broadleaf forest, tropical savannah and open shrubland^46^.

On one hand, these winter areas that we identified matched adequately with previous ringing recoveries for barn swallows breeding in Denmark^47^. On the other hand, we believe that our method to identify potential wintering areas is robust against any bias in ringing effort across Africa. As an example, we identified moult origins in the western portion of South Africa (Fig. 1G), but ringing recoveries in this country are located in the centre and in the east (Fig. 1D), which is in agreement with another study based on NDVI correlation analyses^39^.

### Temporal trends in environmental conditions from wintering areas

**Figure 2:**
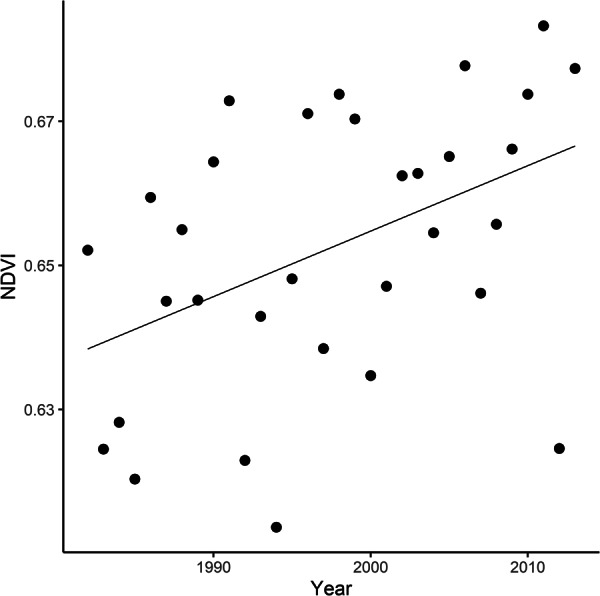
Temporal trend in NDVI extracted from the previously defined wintering areas during the period 1982–2013 (pixels that were assigned to be the likely origin for >60% of our sample; see Fig. 1H). Points represent average NDVI from November (year “i-1”) to March (year “i”). Average NDVI values were obtained from bimonthly data developed by ECOCAST (available at https://ecocast.arc.nasa.gov/data/pub/gimms/3g.v0/). NA pixels within this dataset were filled with package *gapfill*^74^ in R. The continuous line indicates the predicted NDVI values given by the linear model fitted with NDVI as a function of year using Ordinal Least Squares.

Temporal trend in NDVI averaged across all wintering areas increased linearly during the study period (Fig. 2). We found a positive main effect of year on NDVI values extracted from pixels that were assigned to be the likely origin for >60% of our sample (estimate = 0.001, SE = 0.0003, *p* = 0.011; R^2^ = 0.199).

In order to determine differences in environmental conditions within our identified wintering area, we repeated the above analysis for each African biome. Temporal trend in NDVI of savannahs from our wintering area mirrored the average trend found across different biomes (Fig. S1 in Supplementary Materials). Specifically, we found a positive main effect of year and a negative quadratic effect of year on NDVI values extracted from pixels that were assigned to be the likely origin for >50% of our sample in woody savannahs (year: estimate = 0.682, SE = 0.293, *p* = 0.027; year^2^: estimate = −0.0002, SE = 0.0001, *p* = 0.026; R^2^ = 0.493) and savannahs (year: estimate = 0.308, SE = 0.148, *p* = 0.046; year^2^: estimate = −0.0001, SE = 0.0001, *p* = 0.045; R^2^ = 0.365). By contrast, NDVI of evergreen broadleaf forests from our wintering area showed a linear increase through the years (estimate = 0.003, SE = 0.001, *p* = 0.002, R^2^ = 0.268). Finally, NDVI of open shrublands from our wintering area did not show any significant temporal trend (year: estimate = −0.033, SE = 0.085, *p* = 0.701; year^2^: estimate < 0.0001, SE < 0.0001, *p* = 0.7; R^2^ = 0.029).

### Winter conditions *vs* subsequent breeding phenology and reproductive success

**Figure 3:**
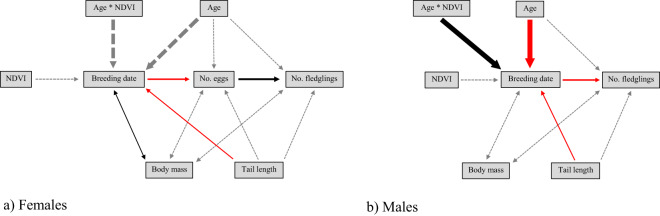
Path diagrams of the causal models developed. One-headed arrows represent a causal effect of one variable on another (i.e. a path). Double-headed arrows link variables with correlated errors. The width of the arrows reflects the magnitude of standardized path coefficients. Black arrows indicate significant positive effects, red arrows significant negative effects and grey arrows non-significant effects.

**Figure 4:**
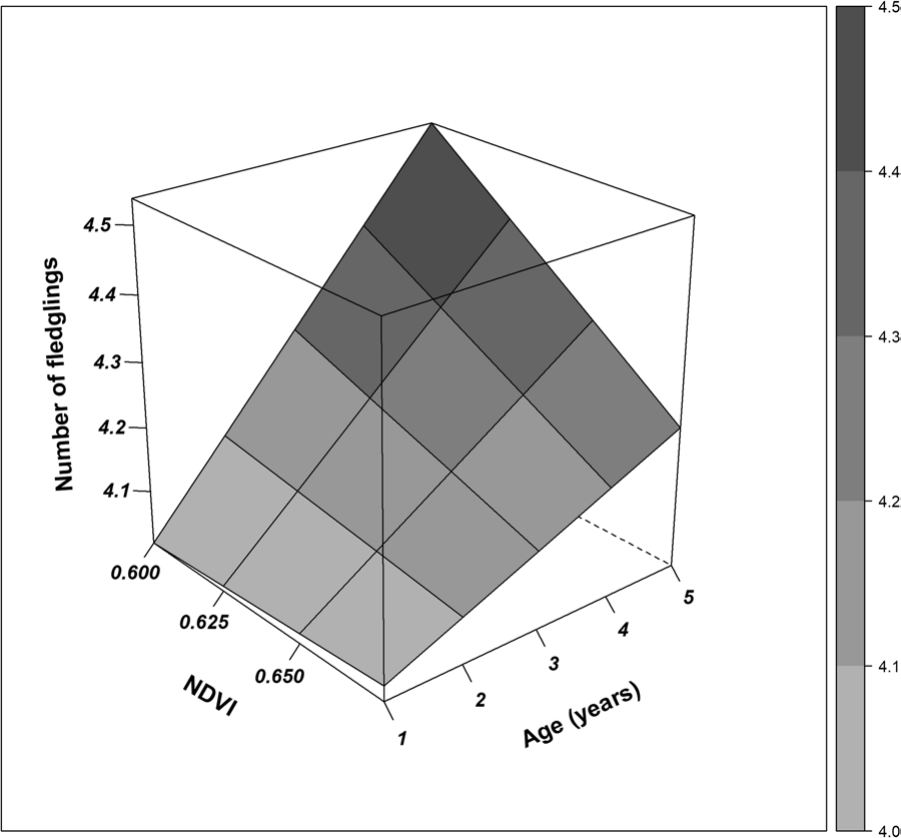
Three-dimensional surface plot showing the relationship between age, NDVI from winter areas and the number of fledglings produced by males at the first brood. Lines and grey scale represent predicted values obtained after calculating the indirect effect of the interaction between age and NDVI on the number of fledglings. This indirect effect was calculated by multiplying standardized path coefficients connecting the interaction term with the final reproductive success.

Environmental conditions from the winter areas carried over to affect subsequent reproductive traits, with effects stronger in males vs females (Table 2; Fig. 3). Confirmatory path analysis revealed that winter conditions affected breeding phenology of males differently according to age class. The direct effect of the interaction term between age and NDVI on breeding date of males was significant [estimate (SE) = 1.849 (0.775), *p* = 0.017]. In addition, breeding date negatively affected the number of fledglings produced by males [estimate (SE) = -0.222 (0.023), *p* < 0.001]. Because both direct effects were significant, the effect of the interaction between age and NDVI on breeding date was ultimately translated to affect the number of fledglings raised by males, and this indirect effect was calculated by multiplying the standardized path coefficients connecting the former and latter variable. Thus, males delayed breeding date (Fig. S2) and produced fewer fledglings (Fig. 4) when NDVI during the previous winter was higher and the slope of this response was increasingly stronger as they aged. For example, considering a shift in NDVI values from bad winter years (i.e. NDVI = 0.6) to good winter years (i.e. NDVI = 0.675), yearling males advanced breeding date only 1 day (from day 35 to day 34), whereas five year-old males delayed breeding date by about 11 days (i.e. from day 21 to day 31). Likewise, for yearling males the path analysis predicted a shift of 0.02 fledglings (i.e. from 4.02 to 4.04) when comparing predicted values from bad winter years to good winter years, whereas for five year-old males, the path analyses predicted a shift of 0.30 fledglings (i.e. from 4.54 to 4.23). Setting winter conditions constant, the shift in breeding date across age was stronger. Specifically, the predicted values obtained for breeding date in response to the age of males (i.e. from 1 to 5 years) accounted for a difference in 14 days (i.e. from day 35 to day 21) under bad winter conditions (i.e. NDVI = 0.6) but the difference in breeding date accounted for only 3 days (i.e. from day 34 to day 31) under good winter conditions (i.e. NDVI = 0.675). Setting winter conditions constant, predicted values obtained for age-related change in the number of fledglings also showed a larger shift. Under bad winter conditions we predicted a change of 0.52 fledglings (i.e. from 4.02 fledglings of one year-old males to 4.54 fledglings of five year-old males), whereas under good winter conditions we predicted a shift of 0.20 fledglings (i.e. from 4.04 fledglings of one year-old males to 4.23 fledglings of five year-old males).

**Table 2:** Summary results from confirmatory path analyses built for each sex class.

Model	Response	Predictor	Estimate	SE	*p*	R^2^
**Females**	**No. fledglings**	**No. eggs**	**0.485**	**0.019**	**<0.001**	0.319
		Age	0.034	0.019	0.076	
		Tail length	0.026	0.019	0.180	
	**No. eggs**	**Breeding date**	**−0.289**	**0.022**	**<0.001**	0.145
		Age	0.003	0.022	0.898	
		Tail length	−0.005	0.021	0.819	
	**Breeding date**	NDVI	−0.075	0.071	0.294	0.196
		Age * NDVI	1.221	0.739	0.100	
		Age	−1.306	0.737	0.078	
		**Tail length**	**−0.059**	**0.022**	**0.007**	
**Males**	**No. fledglings**	**Breeding date**	**−0.222**	**0.023**	**<0.001**	0.167
		Age	0.016	0.022	0.453	
		Tail length	0.020	0.022	0.348	
	**Breeding date**	NDVI	−0.067	0.070	0.345	0.134
		Age * NDVI	**1.849**	**0.775**	**0.017**	
		**Age**	**−1.941**	**0.773**	**0.012**	
		**Tail length**	**−0.095**	**0.022**	**<0.001**	

Estimates shown here are standardized path coefficients (i.e. slopes of effects). R^2^ shown here is the conditional R^2^, based on fixed and random effects. Significant effects are highlighted in bold.

For males, the direct effect of the interaction term between age and NDVI on breeding date was similar to that found for females, but not statistically significant [estimate (SE) = −0.569 (0.339), *p* = 0.094]. In addition, breeding date negatively affected the number of fledglings raised by males [estimate (SE) = −0.222 (0.023), *p* < 0.001]. Consequently, carry-over effects operating in males were similar to that of females, but weaker in strength. Confirmatory path analyses also revaled a significant negative effect of tail length on breeding date for both females [estimate (SE) = −0.060 (0.022), *p* = 0.006] and males [estimate (SE) = −0.097 (0.022), *p* < 0.001]. Therefore, barn swallows with longer tail feathers started to reproduce earlier and ultimately raised more fledglings. In addition, body mass was only significantly correlated with breeding date of females (estimate = 0.042, *p* = 0.029), indicating that females which started to reproduce later were heavier (because most of body mass measures were taken after breeding date).

We used d-separation test to quantify the goodness of fit of our models, which tests the assumption that all variables are conditionally independent^48^. Both path analyses provided robust fit to data (females: Fisher’s C = 15.3, df = 10, *p* = 0.121; males: Fisher’s C = 2.2, df = 4, *p* = 0.699). Thus, we concluded that the hypothesized causal relationships we examined were consistent with the data. By further inspection of “qq plots”, we determined that every single linear mixed model was adequately fitted.

## Discussion

We determined with reasonable precision the wintering areas of barn swallows breeding in Denmark, being distributed from Central to South Africa. This is the first study in which carry-over effects from wintering areas on subsequent reproduction have been analyzed at such large spatial and temporal scales, taking into account age- and sex-dependent variation. Environmental conditions in African wintering areas have improved during the study period. Barn swallows delayed breeding and raised fewer fledglings when experiencing good conditions during the previous winter. Interestingly, we found sex- and age-dependent carry-over effects in the annual cycle from wintering to breeding stage. Environmental conditions experienced during winter affected subsequent reproduction more strongly in males than in females, although both sexes similarly responded to this environmental cue. Individuals of different age responded differently to conditions experienced in winter quarters. In general, there was not a clear response of yearlings to conditions experienced during winter but the response gradually increased as individuals aged. Our results were not confounded by other variables known to affect breeding performance such as body mass and tail length. Finally, the analyses performed were not biased by the spatial scale used to define winter areas.

There is increasing evidence showing that environmental conditions experienced during winter are related to subsequent spring migration, breeding phenology or reproductive success of migratory birds^5,7,9,11,12,17,18,20,38^. Such carry-over effects need to be fully understood because they have important consequences for population dynamics and evolutionary processes^18^. We found that barn swallows delayed breeding date in response to favorable winter conditions, and that individuals responded differently depending on their age. In agreement with our findings, barn swallows breeding in southwestern Spain have been shown to delay arrival to breeding areas in response to good winter conditions^7^, but the opposite outcome was found for barn swallows from northern Italy^38^. In that study, the authors argued that individuals on average acquired body condition earlier during good winter years (i.e. high NDVI) and should depart sooner from winter areas or perform faster migration than during poor winter years (i.e. low NDVI). However, wetter conditions (i.e. high NDVI) may also imply higher abundance of insects and thus a higher probability to be infected with parasites transmitted by mosquitoes, therefore reducing the ability of individuals to initiate early spring migration^7^.

Ecological conditions from the wintering grounds usually affect subsequent breeding success more strongly in females than in males^9,17,18^. Specifically, carry-over effects from winter conditions on timing of breeding and reproductive success were found for female but not for male barn swallows^18^. We found the same carry-over effects operating in both sexes, but the response was stronger for males. A stronger carry-over effect could be expected for females since they must meet the physiological demands associated with egg production. Otherwise, males are expected to arrive earlier than females to breeding grounds because earlier males usually mate sooner and have greater reproductive success^24^. Because competition for early arrival to breeding grounds is higher among the males^13,23^, competition at the wintering habitats could be also higher among the males, which may explain the differences we found between sexes.

It is well known that avian migratory behavior depends on the age of individuals^1,22^. The carry-over effects we found in this study should have been mediated by the effects of different winter conditions on migratory behavior such as departure date from winter areas, flight speed on route, number and duration of stopovers and arrival date to breeding areas. Most previous studies investigating migratory behavior and carry-over effects^5,7,9,11,20^ have discriminated between juveniles *vs* adults and consequently age-related migratory behavior based on actual marked known-age individuals has been reported less frequently (but see Sergio and colaborators^10^). Difference in migratory behavior among age classes is hence poorly known^49–51^.

We found different responses to winter conditions with the age of individuals. Our results support the competition hypothesis and reject the constraint hypotheses to explain age-related differences in timing of reproduction and breeding success. We found the highest difference in reproductive traits across age when winter conditions were unfavorable. Thus, as predicted by the competition hypothesis, if older individuals were dominant over younger ones, greater difference in reproductive traits across age are expected following poor winter conditions. This is because competition among individuals should be exacerbated under low resource availability. Therefore, the higher reproductive success of older individuals compared to yearlings could be due to differences in competence among individuals of different ages that are magnified when experiencing poor winter conditions. Although gaining experience or skills could help older individuals to select better quality wintering habitats or make better use of them than yearlings^27^, in that case, we should expect an improvement in migratory or reproductive performance with age independent of ecological conditions during winter. Our findings are opposite to another study performed with barn swallows in southern Europe, in which experience explained age-related difference in spring migration and reproductive success^6^. However, we acknowledge that we cannot discard the selection hypothesis as a possible explanation in our case.

Our results have implications for conservation and management of migratory insectivorous birds which are declining^52–54^. In this study, we have linked reproductive success of barn swallows breeding in north Europe with environmental changes occurring in the African wintering areas. Furthermore, we have identified that NDVI from woody savannahs and broadleaf forests was the main driver of the general trend in NDVI found for all wintering areas. However, NDVI from savannahs and open scrublands remained stable during the study period. These different trends in ecological conditions on the wintering grounds may help to predict how barn swallows could respond to current global change. Because environmental conditions have remained stable in open savannahs but improved in woody savannahs and rainforests, barn swallows may now encounter more suitable habitats at the latter, which is in agreement with the general northwards shift of their wintering areas^55^. Because African savannahs constitute a highly seasonal environment with periods of heavy rain and periods of severe drought, we can assume that greater rainfall across the wintering areas should be associated with higher values of average NDVI during winter^34^, and furthermore both NDVI and rainfall are positively correlated with abundance of flying insects^31,33,35,36^. Indeed, previous studies have generally assumed that higher NDVI during winter imply favorable ecological conditions for aerial insectivores^5,7,37–39^. Temperature is predicted to increase across Africa, while precipitation is predicted to increase in central Africa and decrease in south Africa^28^. Considering that the correlation of NDVI with these climatic variables differs spatially throughout the winter areas^34^, further complex analyses would be necessary to predict trends in NDVI for these winter areas. Because NDVI has been established as a crucial tool for assessing the effects of climate change on organisms^30^, and specifically migratory birds^5,38^, our results also inform predictions regarding the potential adaptation of migratory aerial insectivores to climate change throughout their life spans.

Summarizing, we identified winter areas of barn swallows breeding in Denmark by combining a multi-isotope assignment with prior information from ringing data. We also determined, using satellite-derived NDVI values, that environmental conditions at the African wintering grounds have improved during our study period. In addition, we have shown that changes in NDVI during winter were related to subsequent reproductive parameters, and that age and sex determined the strength of these carry-over effects. The increasing collection of long-term individual data could shed light on the ability to predict future adaptation of long-distance migrants to current climate change. We highlight that our findings are compiling evidence of the great importance of age on migratory ecology, with relevant fitness consequences.

## Methods

### Field procedures

We monitored 40 breeding colonies of barn swallows at Kraghede (57.22°N, 9.97°E), Denmark, during 1984–2013. This 45 km^2^ study area consists of scattered farms and houses interspersed by meadows and fields where the main crops are wheat and potatoes. APM captured barn swallows with mist nets at least once weekly throughout the breeding season, assuring that more than 95% of all adults are captured and measured annually. All individuals were provided with both metallic and colour rings. We assigned an age of 1 year to all barn swallows when captured the first time as adults. This assumption is supported because breeding dispersal is negligible in the study species. Only three of more than 5000 adults ever moved from one breeding colony to another, and then only a short distance of <750 m^24^. Therefore, we assumed that adult disappearance from the breeding population indicated mortality rather than dispersal^5,7^. Moreover, age assignment was also supported because age at first reproduction was at the age of one year for 100% of recruits. Fieldwork showed that from 315 recruits, all were captured for the first time as an adult when one year old, implying that no recruit was captured the first time when aged two or more years. Therefore, because capture effort was high every year, all recruits without metal rings should be yearlings coming from outside the study sites.

All captured birds were weighed with a Pesola spring balance to the nearest 0.1 g, and tail length was measured to the nearest mm with a ruler. We estimated breeding date of females as the date when they laid the first egg during the first brood, whereas breeding date of males was the date their mate’s laid their first egg. Laying date for females was assigned during weekly visits to nests assuming that one egg was laid daily. Males were assigned to their female patners by regular observations from a hide of colour ringed swallows at their nests using binoculars (8 × 30 Zeiss). We estimated reproductive success as the number of eggs laid and the number of fledglings raised during the first breeding attempt, because we expected that carry-over effects from the winter period would be easily detected during the first part of the breeding stage. Reproductive success at the first brood correlated well with total reproductive success (i.e. including subsequent broods), and so is a good proxy for breeding performance in this multi-brooded species^24^.

During the breeding seasons 2000 and 2016, we collected respectively 84 and 119 feather samples for later stable isotope analyses to identify winter origins. Feather samples from 2000 were taken from the red throat badge, whereas feather samples from 2016 were taken from the rectrix (i.e. outermost feather). Barn swallows usually moult their feathers in the African winter areas^56,57^, and furthermore they moult feathers continuously during almost the whole wintering stay (A. P. Møller, unpublished data from South Africa, Namibia and Ghana). Because feather keratin is metabolically inert after synthesis^58^, we were confident that our feather samples represent the isotopic values derived from the winter areas.

### Stable isotope analysis

All feathers were cleaned of surface oils in 2:1 chloroform:methanol solvent rinse and prepared for *δ*^2^H, *δ*^13^C and *δ*^15^N analysis. Deuterium abundance in the non-exchangeable hydrogen of feathers was determined following standard procedures^59^ and using three calibrated keratin hydrogen-isotope reference materials (CBS = −197‰; SPK = −121.6‰; KHS = −54.1‰). Deuterium measurement was performed on H_2_ gas derived from high-temperature (1350 °C) flash pyrolysis of 350 ± 10 µg feather subsamples and keratin standards. Measurement of the three keratin laboratory reference materials, corrected for linear instrumental drift, were both accurate and precise with typical within-run (n = 5) SD values of <2‰. On the other hand, *δ*^13^C and *δ*^15^N measurements were performed on CO_2_ and N_2_ gases resulted from combustion of 0.5–1.0 feather material. Using previously calibrated internal laboratory C and N standards [powdered keratin (BWBIII; *δ*^13^C = −20‰; *δ*^15^N = 14.4‰) and gelatin (PUGEL; *δ*^13^C = −13.6‰; *δ*^15^N = 4.73‰)], within run (n = 5) precisions for *δ*^15^N and *δ*^13^C measurements were ~ ± 0.15‰. Stable isotope ratios are reported in standard delta (*δ*) notation relative to VSMOW-SLAP (Vienna Standard Mean Ocean Water – Standard Light Antarctic Precipitation) for ^2^H/H, VPDB (Vienna Pee Dee Belemnite carbonate) for ^13^C/^12^C and atmospheric nitrogen for ^15^N/^14^N.

### Combination of ringing recoveries and isotope analysis to identify winter areas

In June 2018, we requested access to all ringing recoveries of Palearctic barn swallows from The European Union for Bird Ringing (EURING). Every ringing scheme, except for the Copenhagen Bird Ringing Centre, agreed to share their data. We originally obtained a sample 2333 individuals that were ringed and recaptured across the barn swallow range^60^. We selected both kinds of recoveries of birds that were either dead or alive. We only retained records of birds captured either in the European breeding areas (from 36° to 66°N and from 9°W to 42°E) during May-August, or in the sub-Saharan wintering range (from 15°N to 35°S and from 17°W to 42°E) during November-February. Finally, because directions followed during migration depend on the longitude of breeding location, we filtered our data subset leaving only individuals whose breeding location was between 5°E and 15°E (i.e. a buffer zone in longitude around Denmark). Following this data filtering, our final sample size for ringing data was 253 individuals ringed in Europe and recaptured in sub-Saharan Africa (or vice versa).

It is well known that ringing effort is very uneven across Africa, being biased to regions of higher density of wintering birds or to more developed countries^40^. This inherent drawback of the banding method for studying migration can be solved by including other sources of information. For instance, ringing recoveries in South Africa indicates that barn swallows breeding in Denmark usually winter in the central and eastern parts of the country, but NDVI correlation analyses point to western South Africa as the main wintering area^39^. This is because ringing effort has been higher at the largest wintering roosts, which are distributed across the centre and east of South Africa, whereas western roosts are smaller and more variably distributed in space and time. Therefore, we considered that combining ringing recoveries with stable isotope analyses would overcome the strong bias in ringing effort; but at the same time, isotope assignment should be constrained longitudinally thanks to ringing recoveries and so the potential moult areas should be identified with higher precision.

To incorporate ringing recoveries within the probabilistic assignment of origins based on istotope analysis, we followed the method described by Van Wilgenburg & Hobson^61^. From our final subset of ringing recoveries (Fig. 1D), first we calculated bearings from breeding to wintering location. Second, we extracted the mean (circular) direction and “kappa” (the concentration parameter, an inverse measure of dispersion). Then, using these parameters, we fit a von Mises probability density function to predict the likelihood of any possible direction (from 1° to 359° separating by 2°). These probability densities were normalized with respect to the sum and were used to create a raster (*fm*) that was used as a prior probability surface (Fig. 1F).

We evaluated the likelihood of origin for a given sample feather by employing a multivariate normal probability density function^62,63^. By applying Bayes’ Rule^61^, we incorporated the prior probability surface (*fm*) in assessing the posterior probabilities of origin (*fx*) based on feather *δ*^2^H, *δ*^13^C and *δ*^15^N. Geographic locations that were consistent with the upper 67% of the spatially explicit probability density maps for each individual (*fx*) were coded as 1; all others were coded as 0^64^. Thus, for each individual being assigned to a moult origin we obtained one binary map, and we depicted the population likely origin by summing over all individual binary surfaces (Fig. 1G). Finally, to select the most-probable wintering areas used by our study populations, we clipped the population likely origin surface at three different threshold values, leaving only pixels that were assigned to be the moult origin for >50, >60 and >70% of our sample (Fig. 1H). We used this cutoff criteria to extract NDVI values at three different spatial scales.

All analyses were conducted in R version 3.3.1^65^ with packages *raster*^66^, *geosphere*^67^, *graticule*^68^, *CircStats*^69^ and *mvnmle*^70^.

### Environmental conditions from wintering areas

We extracted NDVI values for the wintering areas previously identified using the public dataset developed by ECOCAST (available at https://ecocast.arc.nasa.gov/data/pub/gimms/3g.v0/). This data base provided bimonthly NDVI measures at 0.83° resolution, during the period 1981–2013. We used as a proxy of environmental conditions during winter of a given year “i”, the averaged NDVI measures from November (year “i-1”) to March (year “i”). The selection of this time window (November-March) was chosen because this is the average period that barn swallows breeding in Denmark spend wintering in sub-Saharan Africa (A. P. Møller, unpublished data). It has been found that winter areas in southern Africa have moved northwards during 1912–2008 at a rate of 9 km/yr for the Palearctic barn swallow^55^. Consequently, during our study (1984–2013), barn swallow wintering areas may have shifted approximately 290 km northwards.

We were also interested in differences across sub-regions within the identified wintering area. For this purpose, we clipped our wintering area with a land cover classification layer^46^, thus obtaining average NDVI values for each African biome. We used the land cover classification layer from 2011 at 0.1° resolution (available at http://neo.sci.gsfc.nasa.gov/view.php?datasetId=MCD12C1_T1). Because we have inferred the wintering areas of a large population (~ 4,000 individuals) from moult origins given by a much smaller subset (~ 200 sample feathers), in order to test for subsequent fitness carry-over effects we need to use the average NDVI across the whole wintering area. Nevertheless, determining differences in environmental trends within this defined area should provide complementary information to better interpret our results.

### Statistical analysis

To investigate how NDVI from wintering areas may influence subsequent breeding date and reproductive success, we used a structural equation modelling approach. SEMs are probabilistic models that hypothesize a causal network with multiple variables that can appear as both predictor and response variables^71^. We used “confirmatory path analysis” or piecewise SEM, based on applications from graph theory^48^. In piecewise SEM, the causal network is translated to a set of linear equations (e.g. linear mixed models), which are then evaluated individually thus allowing a wide range of distributions and sampling designs. In addition, the goodness of fit of the entire causal network can be quantified by a directed separation test (”d-separation test”), which tests the assumption that all variables are conditionally independent (i.e. that there are no missing relationships among unconnected variables).

We conducted separated confirmatory path analyses for males and females. In this way, different responses across sex are indicated by differences in path coefficients from both causal models. We included eight variables in the model built for females: NDVI from winter areas, age, the interaction term between age and NDVI, tail length, body mass, breeding date, clutch size and number of fledglings in the first brood. For males, we excluded clutch size from the causal model, because this variable should be determined by the female only. Reproductive parameters were considered dependent (endogenous) variables, while the rest were considered independent (exogenous). Every observed variable included in our models had the same sample size (i.e. listwise deletion).

The path analysis built for females was made up of three linear mixed models, and that for males was made up of two linear mixed models (i.e. one for each dependent variable). The structure of the path analyses was designed based on previous knowledge of migratory ecology^17,20^. The first linear model in both path analyses, tested the effects of NDVI from winter areas, age and their interaction term on breeding date, while controlling for the confounding effect of tail length^23,24^. The second linear model for females, tested the effect of breeding date on clutch size while controlling for age and tail length. The third linear model for females tested the effect of clutch size on number of fledglings while controlling for age and tail length. The second linear model for males tested the effect of breeding date on number of fledglings while controlling for age and tail length. Individual identity (ring), breeding colony and year were included as cross-random intercepts in all linear models, thus controlling for repeated measures in the dependent variable taken on the same individual, and also for among breeding site and inter-annual variation. We used normal distributions of errors and the identity link function for all response variables. Residuals of each linear model were visually inspected for deviation from normality using normal qqplots. Finally, we also controlled for the confounding effect of body mass on each reproductive parameter^8^. Our dataset contained measures of body mass before, after or during the first breeding attempt. Thus, in our full dataset, we do not know the direction of causality between body mass and the reproductive parameters. Consequently, to maintain a high sample size, we assumed correlated errors (i.e. a relationship that is bidirectional and assumed to be caused by a shared underlying driver) between body mass and every reproductive parameter.

Finally, we repeated our analyses using the different spatial scales defining wintering areas to determine if spatial scale could influence the results obtained. Thus, we conducted the above described analyses for NDVI extracted at different sizes of wintering areas, as defined by pixels assigned to be the origin for >50, >60 or >70% of our sample. All these analyses performed at different spatial scales provided the same results. Thus, for simplicity, we only present results for NDVI extracted from wintering areas defined as pixels assigned to be the origin for >60% of our sample.

We conducted the confirmatory path analyses with library *piecewiseSEM* ver.1.2.1^71^ and linear mixed models with library *lme4*^72^ using R ver. 3.3.3^65^.

### Ethics statement

Field sampling was conducted in agreement with the national Danish hunting law. Permit for capture and release of birds was obtained from the Danish Ministry of Agriculture. All efforts were made to ameliorate suffering of animals and minimize handling time according to Guidelines to the Use of Wild Birds in Research (J. Fair, E. Paul, and J. Jones, Eds. 2010. Washington, D.C. Ornithological Council).

## Supplementary information

Supplementary Materials

## Data Availability

The dataset analysed during the current study is available from the corresponding author on reasonable request.
