# Author Correction: Age-dependent carry-over effects in a long-distance migratory bird

**DOI:** 10.1038/s41598-021-94870-6

**Published:** 2021-08-11

**Authors:** Cosme López Calderón, Javier Balbontín Arenas, Keith A. Hobson, Anders Pape Møller

**Affiliations:** 1 Department of Zoology, Faculty of Biology, Green Building, Avenue Reina Mercedes, E-41012 Seville, Spain; 2 Department of Biology, University of Western Ontario, London, Ontario Canada; 3 Ecologie Systématique Evolution, Université Paris-Sud, CNRS, AgroParisTech, Université Paris-Saclay, F-91405 Orsay Cedex, France

Correction to: *Scientific Reports* https://doi.org/10.1038/s41598-019-47374-3, published online 19 August 2019

The original version of this Article contained errors. We have detected a technical issue in the dataset we used to obtain Normalized Difference Vegetation Index (NDVI) (available at https://ecocast.arc.nasa.gov/data/pub/gimms/3g.v0/). This has led to a number of changes in interpretations.

Persistent cloud cover or other atmospheric contamination during sensing by the satellite results in dramatically reduced NDVI values at the pixel level^1^. These aberrant NDVI measurements were not present in the dataset we used, rendering “not applicable” (NA) pixels. NA pixels are usually distributed through areas of high cloud cover^2^ (e.g. around the Congo River in our study). If pixels under persistent cloud cover are usually not included in the raster average of a given year, the obtained mean NDVI will be artificially reduced biassing the dataset with pixels of persistent clear sky (typical of drier regions). In addition, we found that the number of NA pixels in each raster average increased through the years (year: estimate = − 18460, SE = 6672, *p* = 0.009; year^2^: estimate = 4.637, SE = 1.67, *p* = 0.009; R^2^ = 0.516), and that the number of NA pixels negatively affected the mean NDVI we calculated for each winter (estimate = − 0.00004, SE = 0.000004, *p* < 0.001; R^2^ = 0.712).

These issues are now solved by applying a gap-filling algorithm to the dataset using the method implemented in the R package *gapfill*^2^*.* We have now re-analysed the data using R versions 3.5.1 and 3.3.3. As a result, the Article changed as follows.

In Abstract,

"Second, we found that environmental conditions as inferred by Normalized Differential Vegetation Index (NDVI) have deteriorated at the wintering grounds. Third, we used confirmatory path analysis to quantify the indirect effect of winter conditions on subsequent breeding success. Females advanced onset of breeding, laid more eggs and raised more fledglings in the first brood when ecological conditions during the previous winter improved. This response was age dependent, since yearlings did not respond to this environmental cue but the response was increasingly stronger as females aged. Males showed a similar response to winter conditions, although not statistically significant."

now reads

"Second, we found that environmental conditions as inferred by Normalized Difference Vegetation Index (NDVI) have improved at the wintering grounds. Third, we used confirmatory path analysis to quantify the indirect effect of winter conditions on subsequent breeding success. Males delayed onset of breeding and raised fewer fledglings in the first brood when ecological conditions during the previous winter improved. This response was age dependent, since yearlings did not respond to this environmental cue but the response was increasingly stronger as males aged. Females showed a similar response to winter conditions, although not statistically significant."

In Results, under subheading ‘Temporal trends in environmental conditions from wintering areas’,

“Temporal trend in NDVI averaged across all the wintering area showed a decline after 1991 (Figure 2). We found a positive main effect of year and a negative quadratic effect of year on NDVI values extracted from pixels that were assigned to be the likely origin for > 50% of our sample (year: estimate = 0.747, SE = 0.359, *p* = 0.046; year^2^: estimate = − 0.0002, SE = 0.0001, *p* = 0.046; R^2^ = 0.189), > 60% of our sample (year: estimate = 0.829, SE = 0.386, *p* = 0.040; year^2^: estimate = − 0.0002, SE = 0.0001, *p* = 0.040; R^2^ = 0.211) and 70% of our sample (year: estimate = 0.879, SE = 0.393, *p* = 0.033; year^2^: estimate = − 0.0002, SE = 0.0001, *p* = 0.033; R^2^ = 0.2).

In order to determine differences in environmental conditions within our identified wintering area, we repeated the above analysis for each African biome. Temporal trend in NDVI of savannahs from our wintering area mirrored the average trend found across different biomes (Figure S1 in Supplementary Materials). Specifically, we found a positive main effect of year and a negative quadratic effect of year on NDVI values extracted from pixels that were assigned to be the likely origin for > 50% of our sample in woody savannahs (year: estimate = 0.682, SE = 0.293, *p* = 0.027; year^2^: estimate = − 0.0002, SE = 0.0001, *p* = 0.026; R^2^ = 0.493) and savannahs (year: estimate = 0.308, SE = 0.148, *p* = 0.046; year^2^: estimate = − 0.0001, SE = 0.0001, *p* = 0.045; R^2^ = 0.365). By contrast, NDVI of evergreen broadleaf forests from our wintering area showed a linear increase through the years (estimate = 0.003, SE = 0.001, *p* = 0.002, R^2^ = 0.268). Finally, NDVI of open shrublands from our wintering area did not show any significant temporal trend (year: estimate = − 0.033, SE = 0.085, *p* = 0.701; year^2^: estimate < 0.0001, SE < 0.0001, *p* = 0.7; R^2^ = 0.029)."

now reads

“Temporal trend in NDVI averaged across all wintering areas increased linearly during the study period (Figure 2). We found a positive main effect of year on NDVI values extracted from pixels that were assigned to be the likely origin for > 60% of our sample (estimate = 0.001, SE = 0.0003, *p* = 0.011; R^2^ = 0.199).

In order to determine differences in environmental conditions within our identified wintering area, we repeated the above analysis for each African biome. Temporal trend in NDVI of woody savannahs and evergreen broadleaf forest mirrored the average trend found across different biomes (Figure S1). Specifically, we found a positive main effect of year on NDVI values extracted from pixels that were assigned to be the likely origin for > 60% of our sample in woody savannahs (year: estimate = 0.001, SE = 0.0003, *p* = 0.005; R^2^ = 0.235) and broadleaf forest (year: estimate = 0.001, SE = 0.0005, *p* = 0.022; R^2^ = 0.164). Finally, NDVI of open shrublands (estimate = 0.0002, SE = 0.0001, *p* = 0.107; R^2^ = 0.084) and savannahs (estimate = 0.0001, SE = 0.0003, *p* = 0.756; R^2^ = 0.003) from our wintering area did not show any significant temporal trend."

In Results, under subheading ‘Winter conditions *vs* subsequent breeding phenology and reproductive success’,

“Environmental conditions from the winter areas carried over to affect subsequent reproductive traits, with effects stronger in females compared to males (Table 2; Figure 3). Confirmatory path analysis revealed that winter conditions affected breeding phenology of females differently according to age class. The direct effect of the interaction term between age and NDVI on breeding date of females was significant [estimate (SE) = − 0.712 (0.323), *p* = 0.028]. In addition, breeding date negatively affected clutch size of females [estimate (SE) = − 0.289 (0.022), *p* < 0.001]. Furthermore, clutch size positively affected the number of fledglings produced by females [estimate (SE) = 0.484 (0.019), *p* < 0.001]. Because all these direct effects were significant, the effect of the interaction between age and NDVI on breeding date was ultimately translated to affect the number of fledglings raised, and this indirect effect was calculated by multiplying the standardized path coefficients connecting the former and latter variable. Thus, females advanced laying date (Figure S2), laid more eggs (Figure S3) and produced more fledglings (Figure 4) when NDVI during the previous winter was higher and the slope of this response was increasingly stronger as they aged. For example, considering a shift on NDVI values from bad winter years (i.e. NDVI = 0.5) to good winter years (i.e. NDVI = 0.7), yearling females advanced breeding date only 3 days (from day 144 to day 141), whereas 5 year-old females advanced breeding date by about 18 days (i.e. from day 108 to day 90). Likewise, for yearling females the path analysis predicted a shift of 0.07 eggs (i.e. from 4.95 to 5.02) and 0.06 fledglings (i.e., from 4.12 to 4.18) when comparing predicted values from bad winter years to good winter years, whereas for 5 year-old females the path analyses predicted a shift of 0.34 eggs (i.e., from 5.64 to 5.98) and 0.32 fledglings (i.e., from 4.76 to 5.09). On the other hand, setting winter conditions constant, the shift in breeding date across age was much stronger. Specifically, the predicted values obtained for breeding date in response to the age of females (i.e. from 1 to 5 years) accounted for a difference in 36 days (i.e., from day 144 to day 108) under bad winter conditions (i.e. NDVI = 0.5), whereas the difference in breeding date accounted for 50 days (i.e., from day 140 to day 90) under good winter conditions (i.e. NDVI = 0.7). Likewise, setting winter conditions constant, predicted values obtained for age-related change in clutch size and number of fledglings also showed a large shift. Specifically, under bad winter conditions our analyses predicted a change of 0.68 eggs (i.e., from 4.95 eggs of 1 year-old females to 5.63 eggs of 5 year-old females) and 0.65 fledglings (i.e., from 4.12 fledglings of 1 year-old females to 4.77 fledglings of 5 year-old females), whereas under good winter conditions our analyses predicted a larger change of 1 egg (i.e., from 5.02 eggs of 1 year-old females to 5.98 eggs of 5 year-old females) and 1 fledgling (i.e., from 4.18 fledglings of 1 year-old females to 5.09 fledglings of 5 year-old females).

For males, the direct effect of the interaction term between age and NDVI on breeding date was similar to that found for females, but not statistically significant [estimate (SE) = − 0.569 (0.339), *p* = 0.094]. In addition, breeding date negatively affected the number of fledglings raised by males [estimate (SE) = − 0.222 (0.023), *p* < 0.001]. Consequently, carry-over effects operating in males were similar to that of females, but weaker in strength. Confirmatory path analyses also revealed a significant negative effect of tail length on breeding date for both females [estimate (SE) = − 0.060 (0.022), *p* = 0.006] and males [estimate (SE) = − 0.097 (0.022), *p* < 0.001]. Therefore, barn swallows with longer tail feathers started to reproduce earlier and ultimately raised more fledglings. In addition, body mass was only significantly correlated with breeding date of females (estimate = 0.042, *p* = 0.029), indicating that females which started to reproduce later were heavier (because most of body mass measures were taken after breeding date).

We used d-separation test to quantify the goodness of fit of our models, which tests the assumption that all variables are conditionally independent^48^. Both path analyses provided robust fit to data (females: Fisher’s C = 15.3, df = 10, *p* = 0.121; males: Fisher’s C = 2.2, df = 4, *p* = 0.699). Thus, we concluded that the hypothesized causal relationships we examined were consistent with the data. By further inspection of “qq plots”, we determined that every single linear mixed model was adequately fitted."

now reads

“Environmental conditions from the winter areas carried over to affect subsequent reproductive traits, with effects stronger in males vs. females (Table 2; Figure 3). Confirmatory path analysis revealed that winter conditions affected breeding phenology of males differently according to age class. The direct effect of the interaction term between age and NDVI on breeding date of males was significant [estimate (SE) = 1.849 (0.775), *p* = 0.017]. In addition, breeding date negatively affected the number of fledglings produced by males [estimate (SE) = − 0.222 (0.023), *p* < 0.001]. Because both direct effects were significant, the effect of the interaction between age and NDVI on breeding date was ultimately translated to affect the number of fledglings raised by males, and this indirect effect was calculated by multiplying the standardized path coefficients connecting the former and latter variable. Thus, males delayed breeding date (Figure S2) and produced fewer fledglings (Figure 4) when NDVI during the previous winter was higher and the slope of this response was increasingly stronger as they aged. For example, considering a shift in NDVI values from bad winter years (i.e. NDVI = 0.6) to good winter years (i.e. NDVI = 0.675), yearling males advanced breeding date only 1 day (from day 35 to day 34), whereas 5 year-old males delayed breeding date by about 11 days (i.e. from day 21 to day 31). Likewise, for yearling males the path analysis predicted a shift of 0.02 fledglings (i.e. from 4.02 to 4.04) when comparing predicted values from bad winter years to good winter years, whereas for 5 year-old males, the path analyses predicted a shift of 0.30 fledglings (i.e. from 4.54 to 4.23). Setting winter conditions constant, the shift in breeding date across age was stronger. Specifically, the predicted values obtained for breeding date in response to the age of males (i.e. from 1 to 5 years) accounted for a difference in 14 days (i.e. from day 35 to day 21) under bad winter conditions (i.e. NDVI = 0.6) but the difference in breeding date accounted for only 3 days (i.e. from day 34 to day 31) under good winter conditions (i.e. NDVI = 0.675). Setting winter conditions constant, predicted values obtained for age-related change in the number of fledglings also showed a larger shift. Under bad winter conditions we predicted a change of 0.52 fledglings (i.e. from 4.02 fledglings of 1 year-old males to 4.54 fledglings of 5 year-old males), whereas under good winter conditions we predicted a shift of 0.20 fledglings (i.e. from 4.04 fledglings of 1 year-old males to 4.23 fledglings of 5 year-old males).

For females, the direct effect of the interaction term between age and NDVI on breeding date was similar to that found for males, but not statistically significant [estimate (SE) = 1.221 (0.739), *p* = 0.100]. In addition, breeding date negatively affected the number of eggs laid [estimate (SE) = − 0.289 (0.022), *p* < 0.001], and this in turn affected the number of fledglings raised by females [estimate (SE) = 0.485 (0.019), *p* < 0.001]. Consequently, carry-over effects operating in females were similar to that of males, but weaker in strength. Confirmatory path analyses also revealed a significant negative effect of tail length on breeding date for both females [estimate (SE) = − 0.059 (0.022), *p* = 0.007] and males [estimate (SE) = − 0.095 (0.022), *p* < 0.001]. Therefore, barn swallows with longer tail feathers started to reproduce earlier and ultimately raised more fledglings. In addition, body mass was only significantly correlated with breeding date of females (estimate = 0.04, *p* = 0.035), indicating that females who started to reproduce later were heavier (because most of body mass measures were taken after breeding date).

We used d-separation test to quantify the goodness of fit of our models, a procedure that tests the assumption that all variables are conditionally independent^48^. Both path analyses provided robust fit to data (females: Fisher’s C = 10.87, df = 10, *p* = 0.367; males: Fisher’s C = 0.37, df = 4, *p* = 0.985). Thus, we concluded that the hypothesized causal relationships we examined were consistent with the data. By further inspection of “qq plots”, we determined that every single linear mixed model was adequately fitted."

In Discussion,

"We determined with reasonable precision the wintering areas of barn swallows breeding in Denmark, being distributed from Central to South Africa. This is the first study in which carry-over effects from wintering areas on subsequent reproduction have been analyzed at such large spatial and temporal scales, taking into account age- and sex-dependent variation. Environmental conditions in African wintering areas have changed during the study period showing a steep decline after 1991. We found that barn swallows advanced breeding date, produced larger clutches and raised more fledglings when experiencing good conditions during the previous winter. Interestingly, we found sex- and age-dependent carry-over effects in the annual cycle from wintering to breeding stage. Environmental conditions experienced during winter affected subsequent reproduction more strongly in females than in males, although both sexes similarly responded to this environmental cue. Individuals of different age responded differently to conditions experienced in winter quarters. In general, there was not a clear response of yearlings to conditions experienced during winter, whereas the response gradually increased as individuals aged. Additionally, our results were not confounded by other variables known to affect breeding performance, such as body mass and tail length. Finally, the analyses performed were not biased by the spatial scale used to define winter areas.

There is increasing evidence showing that environmental conditions experienced during winter are related to subsequent spring migration, breeding phenology or reproductive success of migratory birds^5,7,9,11,12,17,18,20,38^. Such carry-over effects need to be fully understood because they have important consequences for population dynamics and evolutionary processes^18^. We found that barn swallows advanced breeding date in response to favorable winter conditions, and that individuals responded differently depending on their age. In agreement with our findings, barn swallows breeding in northern Italy have also been shown to advance arrival and breeding in response to good winter conditions when studied at the population level^5,38^, but the opposite outcome was found at the individual level^18^. These authors argued that when studied at the population level, individuals on average acquired body condition earlier during good winter years (i.e. high NDVI) and should depart sooner from winter areas or perform faster migration than during poor winter years (i.e. low NDVI). However, when studied at the individual level, individuals may experience different winter conditions within years and those individuals that experienced poor winter conditions (i.e. low NDVI) should depart earlier from winter areas because of seasonal deterioration of winter conditions taking place within years^18^. Therefore, both spatial and temporal variation in winter conditions could influence carry-over effects between the wintering and breeding stage. Hence, the outcome could differ when the same population of the same species is studied either at the individual or population level.

Ecological conditions from the wintering grounds usually affect subsequent breeding success more strongly in females than in males^9,17,18^. Specifically, carry-over effects from winter conditions on timing of breeding and reproductive success were found for female but not for male barn swallows^18^. We found the same carry-over effects operating in both sexes, but in agreement with the latter study, the response was stronger for females. This difference found between sexes could be explained because females may be more constrained than males in the time needed to start breeding after arrival to their breeding grounds. Along this line, the variance in time elapsed between arrival and breeding was smaller for females compared to males^18^. Alternatively, the acquisition of an appropriate physical condition after arrival to breeding areas could be more important to determine breeding date for females than for males, because females must meet the physiological demands associated with egg production. Otherwise, males are more prone to arrive earlier than females to breeding grounds because males that arrive earlier usually mate sooner and have greater reproductive success^24^. Because we lacked arrival date in our path analyses, we could not make many inference about the causes of small difference we found in carry over effects between sexes.

It is well known that avian migratory behavior depends on the age of individuals^1,22]^. The carry-over effects we found in this study should have been mediated by the effects of different winter conditions on migratory behavior such as departure date from winter areas, flight speed on route, number and duration of stopovers and arrival date to breeding areas. Most previous studies investigating migratory behavior and carry-over effects^5,7,9,11,20^ have discriminated between two age classes (i.e. juveniles vs. adults) and consequently age-related migratory behavior based on actual known age marked-individuals has been reported less frequently (but see Sergio and colaborators^10^). Difference amongst age classes in migratory behavior is hence a less studied phenomenon^49–51^.

We found different responses to winter conditions with the age of individuals. Our results support the constraint hypothesis and reject the competition hypotheses to explain age-related differences in timing of reproduction and breeding success. We found the highest difference in reproductive traits across age when winter conditions were favorable. If the competition hypothesis was operating in our study system and if older individuals were dominant over young ones, greater difference in reproductive traits across age were expected after experiencing poor winter conditions. This is because competition among individuals should be exacerbated under low resource availability. However, we found the opposite, and we hypothesize that the higher reproductive success of older individuals compared to yearlings could be due to swallows acquiring competence via experience. For instance, gaining experience or skills could help older individuals to select better quality wintering habitats or make better use of them than yearlings^27^. Our findings are consistent with another study performed with barn swallows in southern Europe, in which experience explained age-related difference in spring migration and reproductive success^6^. However, we acknowledge that we cannot discard the selection hypothesis as a possible explanation in our case.

Our results have implications for conservation and management of migratory insectivorous birds which are declining^52–54^. In this study, we have linked reproductive success of barn swallows breeding in north Europe with environmental changes occurring in the African wintering areas. Furthermore, we have identified that NDVI from savannahs was the main driver of the general trend in NDVI found for all wintering areas. We suggest that the dramatic decline in NDVI we found for woody savannahs could be buffered by the increase in equatorial broadleaf forests. These different trends in ecological conditions within the wintering areas may help to predict how barn swallows could respond to current global change. Because environmental conditions have deteriorated in savannahs and improved in rainforests, barn swallows may now encounter more suitable habitats at the latter, which is in agreement with the general northwards shift of their wintering areas^55^. Because African savannahs constitute a highly seasonal environment with periods of heavy rain and periods of severe drought, we can assume that greater rainfall across the wintering areas should be associated with higher values of average NDVI during winter^34^, and furthermore both NDVI and rainfall are positively correlated with abundance of flying insects^31,33,35,36^. Indeed, previous studies have generally assumed that higher NDVI during winter imply favorable ecological conditions for aerial insectivores to overwinter^5,7,37–39^. Temperature is predicted to increase across Africa, while precipitation is predicted to increase in central Africa and decrease in south Africa^28^. Considering that the correlation of NDVI with these climatic variables differs spatially throughout the winter areas^34^, further complex analyses would be necessary to predict trends in NDVI for these winter areas. Because NDVI has been established as a crucial tool for assessing the effects of climate change on organisms^30^, and specifically migratory birds^5,38^, our results are also appropriate for understanding and predicting the potential adaptation to climate change of organisms throughout their life spans.

Summarizing, we identified winter areas of barn swallows breeding in Denmark by combining a multi-isotope assignment with prior information from ringing data. We have also determined, using satellite-derived NDVI values, that environmental conditions at the African wintering grounds have deteriorated in recent years. In addition, we have shown that changes in NDVI during winter were related to subsequent reproductive parameters, and that age and sex determined the strength of these carry-over effects. The increasing collection of long-term individual data could shed light on the ability to predict future adaptation of long-distance migrants to current climate change. We highlight that our findings are compiling evidence of the great importance of age on migratory ecology, with relevant fitness consequences."

now reads

"We determined with reasonable precision the wintering areas of barn swallows breeding in Denmark, being distributed from Central to South Africa. This is the first study in which carry-over effects from wintering areas on subsequent reproduction have been analyzed at such large spatial and temporal scales, taking into account age- and sex-dependent variation. Environmental conditions in African wintering areas have improved during the study period. Barn swallows delayed breeding and raised fewer fledglings when experiencing good conditions during the previous winter. Interestingly, we found sex- and age-dependent carry-over effects in the annual cycle from wintering to breeding stage. Environmental conditions experienced during winter affected subsequent reproduction more strongly in males than in females, although both sexes similarly responded to this environmental cue. Individuals of different age responded differently to conditions experienced in winter quarters. In general, there was not a clear response of yearlings to conditions experienced during winter but the response gradually increased as individuals aged. Our results were not confounded by other variables known to affect breeding performance such as body mass and tail length. Finally, the analyses performed were not biased by the spatial scale used to define winter areas.

There is increasing evidence showing that environmental conditions experienced during winter are related to subsequent spring migration, breeding phenology or reproductive success of migratory birds^5,7,9,11,12,17,18,20,38^. Such carry-over effects need to be fully understood because they have important consequences for population dynamics and evolutionary processes^18^. We found that barn swallows delayed breeding date in response to favorable winter conditions, and that individuals responded differently depending on their age. In agreement with our findings, barn swallows breeding in southwestern Spain have been shown to delay arrival to breeding areas in response to good winter conditions^7^, but the opposite outcome was found for barn swallows from northern Italy^38^. In that study, the authors argued that individuals on average acquired body condition earlier during good winter years (i.e. high NDVI) and should depart sooner from winter areas or perform faster migration than during poor winter years (i.e. low NDVI). However, wetter conditions (i.e. high NDVI) may also imply higher abundance of insects and thus a higher probability to be infected with parasites transmitted by mosquitoes, therefore reducing the ability of individuals to initiate early spring migration^7^.

Ecological conditions from the wintering grounds usually affect subsequent breeding success more strongly in females than in males^9,17,18^. Specifically, carry-over effects from winter conditions on timing of breeding and reproductive success were found for female but not for male barn swallows^18^. We found the same carry-over effects operating in both sexes, but the response was stronger for males. A stronger carry-over effect could be expected for females since they must meet the physiological demands associated with egg production. Otherwise, males are expected to arrive earlier than females to breeding grounds because earlier males usually mate sooner and have greater reproductive success^24^. Because competition for early arrival to breeding grounds is higher among the males^13,23^, competition at the wintering habitats could be also higher among the males, which may explain the differences we found between sexes.

It is well known that avian migratory behavior depends on the age of individuals^1,22]^. The carry-over effects we found in this study should have been mediated by the effects of different winter conditions on migratory behavior such as departure date from winter areas, flight speed on route, number and duration of stopovers and arrival date to breeding areas. Most previous studies investigating migratory behavior and carry-over effects^5,7,9,11,20^ have discriminated between juveniles vs. adults and consequently age-related migratory behavior based on actual marked known-age individuals has been reported much less frequently (but see Sergio and colaborators^10^). Difference in migratory behavior among age classes is hence poorly known^49–51^.

We found different responses to winter conditions with the age of individuals. Our results support the competition hypothesis and reject the constraint hypotheses to explain age-related differences in timing of reproduction and breeding success. We found the highest difference in reproductive traits across age when winter conditions were unfavorable. Thus, as predicted by the competition hypothesis, if older individuals were dominant over younger ones, greater difference in reproductive traits across age are expected following poor winter conditions. This is because competition among individuals should be exacerbated under low resource availability. Therefore, the higher reproductive success of older individuals compared to yearlings could be due to differences in competence among individuals of different ages that are magnified when experiencing poor winter conditions. Although gaining experience or skills could help older individuals to select better quality wintering habitats or make better use of them than yearlings^27^, in that case, we should expect an improvement in migratory or reproductive performance with age independent of ecological conditions during winter. Our findings are opposite to another study performed with barn swallows in southern Europe, in which experience explained age-related difference in spring migration and reproductive success^6^. However, we acknowledge that we cannot discard the selection hypothesis as a possible explanation in our case.

Our results have implications for conservation and management of migratory insectivorous birds which are declining^52–54^. In this study, we have linked reproductive success of barn swallows breeding in north Europe with environmental changes occurring in the African wintering areas. Furthermore, we have identified that NDVI from woody savannahs and broadleaf forests was the main driver of the general trend in NDVI found for all wintering areas. However, NDVI from savannahs and open scrublands remained stable during the study period. These different trends in ecological conditions on the wintering grounds may help to predict how barn swallows could respond to current global change. Because environmental conditions have remained stable in open savannahs but improved in woody savannahs and rainforests, barn swallows may now encounter more suitable habitats at the latter, which is in agreement with the general northwards shift of their wintering areas^55^. Because African savannahs constitute a highly seasonal environment with periods of heavy rain and periods of severe drought, we can assume that greater rainfall across the wintering areas should be associated with higher values of average NDVI during winter^34^, and furthermore both NDVI and rainfall are positively correlated with abundance of flying insects^31,33,35,36^. Indeed, previous studies have generally assumed that higher NDVI during winter imply favorable ecological conditions for aerial insectivores^5,7,37,38,39^. Temperature is predicted to increase across Africa, while precipitation is predicted to increase in central Africa and decrease in south Africa^28^. Considering that the correlation of NDVI with these climatic variables differs spatially throughout the winter areas^34^, further complex analyses would be necessary to predict trends in NDVI for these winter areas. Because NDVI has been established as a crucial tool for assessing the effects of climate change on organisms^30^, and specifically migratory birds^5,38^, our results also inform predictions regarding the potential adaptation of migratory aerial insectivores to climate change throughout their life spans.

Summarizing, we identified winter areas of barn swallows breeding in Denmark by combining a multi-isotope assignment with prior information from ringing data. We also determined, using satellite-derived NDVI values, that environmental conditions at the African wintering grounds have improved during our study period. In addition, we have shown that changes in NDVI during winter were related to subsequent reproductive parameters, and that age and sex determined the strength of these carry-over effects. The increasing collection of long-term individual data could shed light on the ability to predict future adaptation of long-distance migrants to current climate change. We highlight that our findings are compiling evidence of the great importance of age on migratory ecology, with relevant fitness consequences."

Additionally, Figures. 2, 3, and 4, as well as legends for Figures. 2 and 4 have been corrected. The original Figures 2, 3, and 4, and original legends for Figures 2 and 4, are reproduced below for the record.

**Figure 2:**
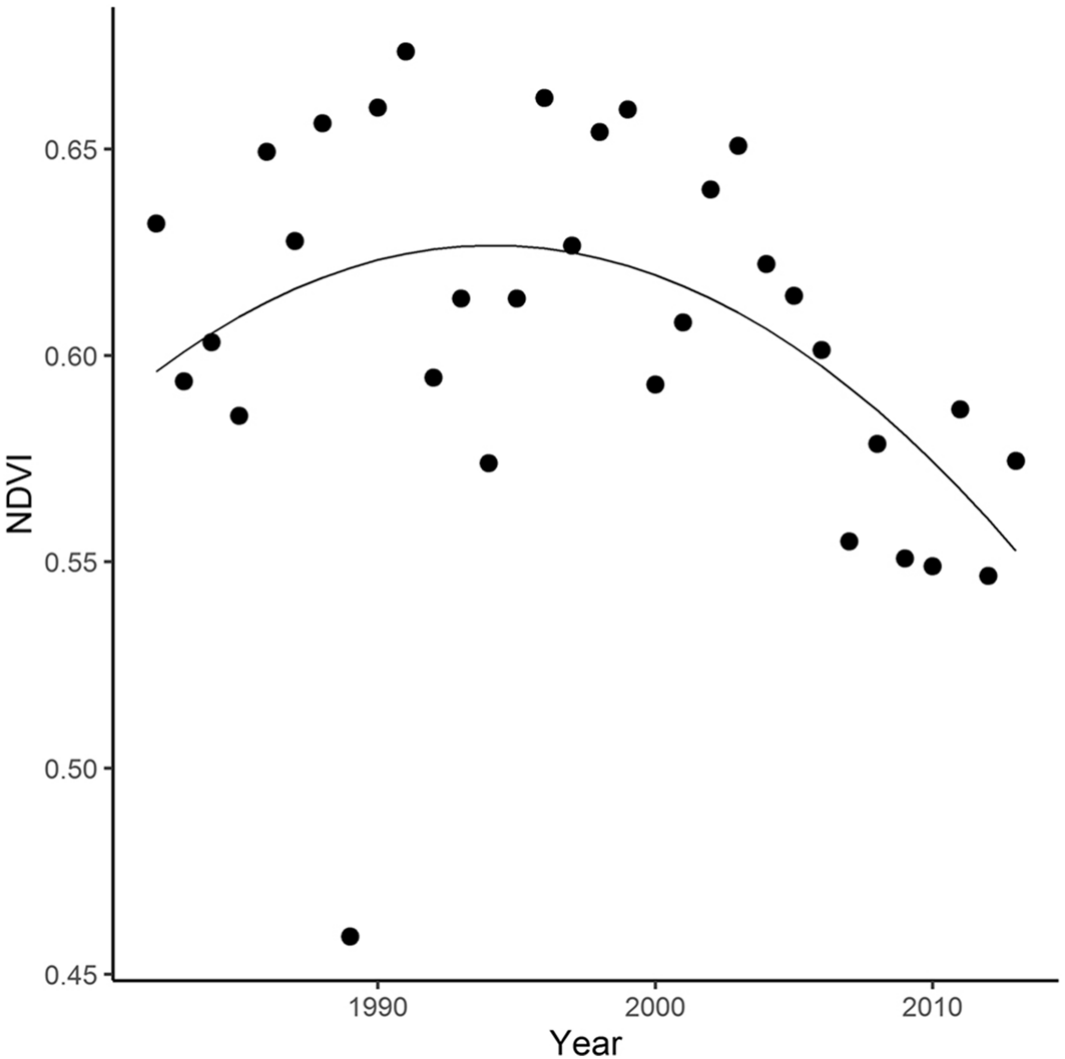
Temporal trend in NDVI extracted from the previously defined wintering areas during the period 1981–2013 (pixels that were assigned to be the likely origin for > 60% of our sample; see Figure. 1H). Points represent average NDVI from November (year “i-1”) to March (year “i”). Average NDVI values were obtained from bimonthly data developed by ECOCAST (available at https://ecocast.arc.nasa.gov/data/pub/gimms/3g.v0/). The continuous line indicates the predicted NDVI values given by the linear model fitted with NDVI as a function of year and second degree of year using Ordinal Least Squares.

**Figure 3:**
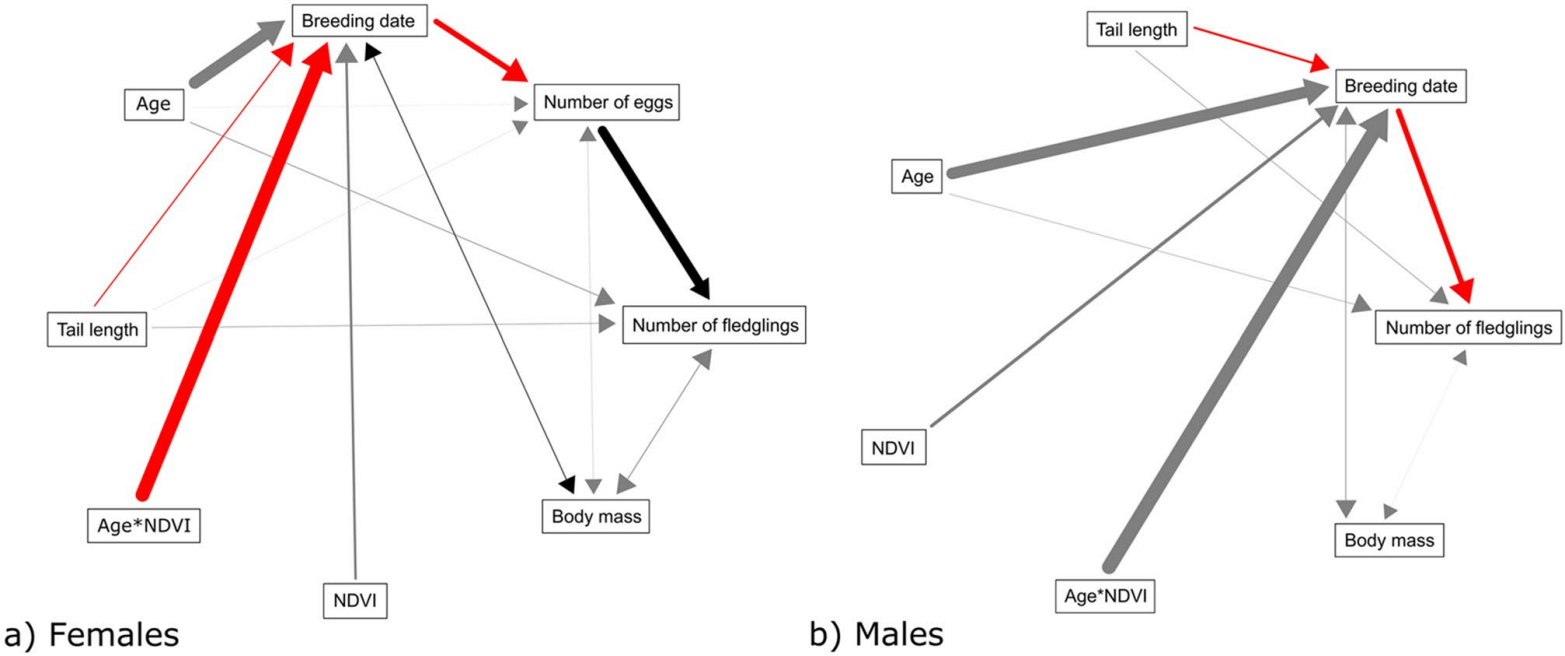
The original version of Figure 3.

**Figure 4:**
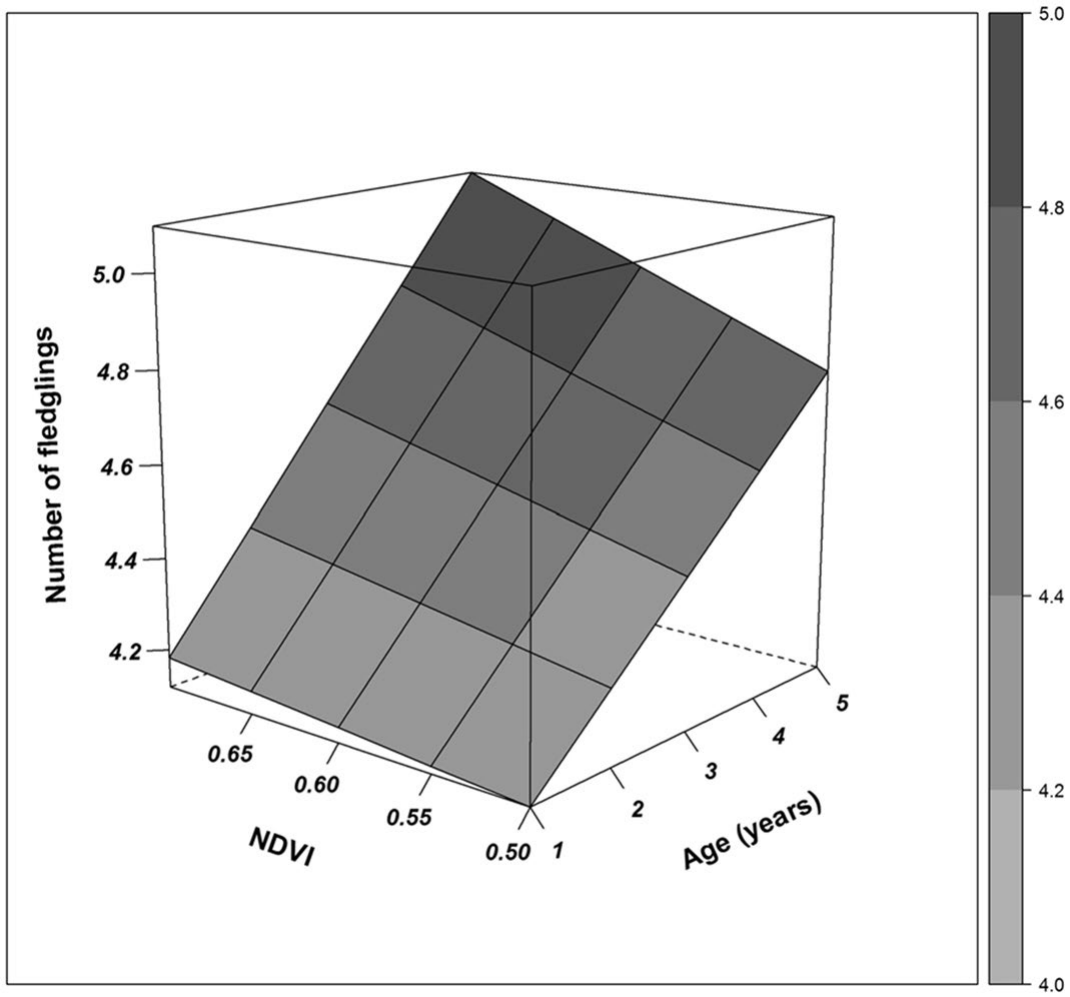
Three-dimensional surface plot showing the relationship between age, NDVI from winter areas and the number of fledglings produced by females at the first brood. Lines and grey scale represent predicted values obtained after calculating the indirect effect of the interaction between age and NDVI on the number of fledglings. This indirect effect was calculated by multiplying standardized path coefficients connecting the interaction term with the final reproductive success.

Table 2 has also been corrected. The original version of this Table is reproduced below for reference:

**Table 2:** Summary results from confirmatory path analyses built for each sex class.

Model	Response	Predictor	Estimate	SE	*p*	R^2^
Females	**No. fledglings**	**No. eggs**	**0.484**	**0.019**	**< 0.001**	0.318
		Age	0.036	0.019	0.066	
		Tail lenght	0.026	0.019	0.179	
	**No. eggs**	**Breeding date**	**− 0.289**	**0.022**	**< 0.001**	0.142
		Tail	− 0.005	0.021	0.826	
		Age	0.003	0.022	0.879	
	**Breeding date**	**Tail lenght**	**− 0.060**	**0.022**	**0.006**	0.197
		Age * NDVI	**− 0.712**	**0.323**	**0.028**	
		Age	0.617	0.322	0.056	
		NDVI	0.125	0.081	0.129	
Males	**No. fledglings**	**Breeding date**	**− 0.222**	**0.023**	**< 0.001**	0.167
		Tail lenght	0.020	0.022	0.348	
		Age	0.016	0.022	0.456	
	**Breeding date**	**Tail lenght**	**− 0.097**	**0.022**	**< 0.001**	0.134
		NDVI	0.134	0.078	0.092	
		Age * NDVI	− 0.569	0.339	0.094	
		Age	0.466	0.336	0.167	

Estimates shown here are standardized path coefficients (i.e. slopes of effects). R^2^ shown here is the conditional R^2^, based on fixed and random effects. Significant effects are highlighted in bold.

Finally, Supplementary Figures S1 and S2 have been corrected. The original Supplementary Information file is appended to this notice for reference.

The original Article and accompanying Supplementary Information file have been corrected.

## Supplementary Information

Supplementary Information.
