# Supplementary material for: Age-dependent carry-over effects in a long-distance migratory bird
Source: Sci Rep. 2019 Aug 19;9:12032. doi: 10.1038/s41598-019-47374-3 (PMC6700079; doi:10.1038/s41598-019-47374-3)
Supplement: Supplementary file 1 — Supplementary Materials [file 41598_2019_47374_MOESM1_ESM.docx]

**Supplementary Materials:**

**Age-dependent carry-over effects in a long distance migratory bird**

Cosme López Calderón^1^, Javier Balbontín Arenas^1^, Keith A. Hobson^2^ and Anders Pape Møller^3^

^1^*Department of Zoology, Faculty of Biology, Green Building, Avenue Reina Mercedes, E-41012 Seville, Spain*

^2^ *Department of Biology, University of Western Ontario, London, Ontario, Canada*

^3^ *Ecologie Systématique Evolution, Université Paris-Sud, CNRS, AgroParisTech, Université Paris-Saclay, F-91405 Orsay Cedex, France*

Correspondence to CLC:

Tel: (+34) 617 71 30 54

E-mail: clopez25@us.es

**
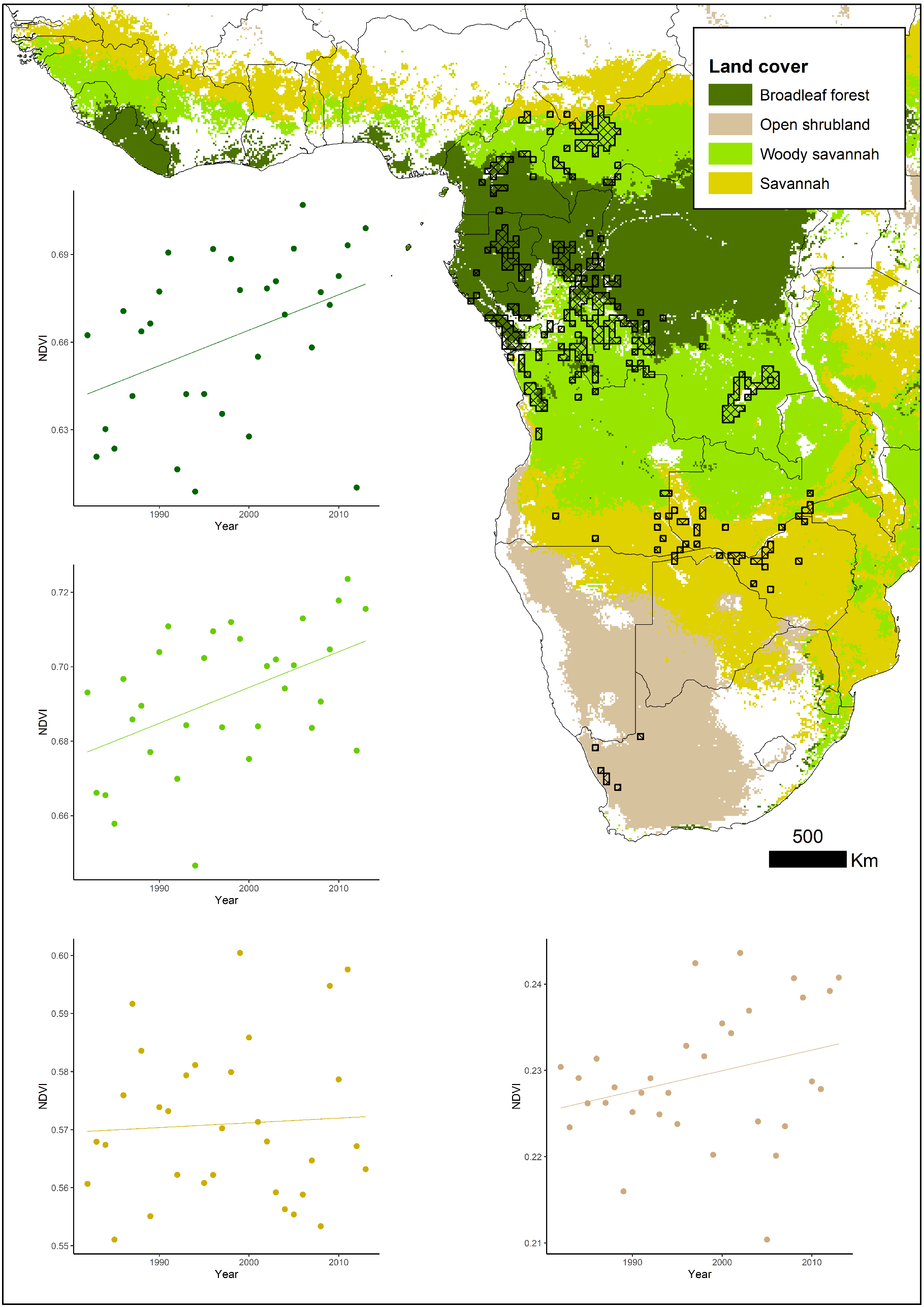
**

**Figure S1.** Temporal trends in NDVI across African biomes from our identified wintering areas. Pixels that were assigned to be the likely origin for >60% of our sample were determined by the grid (outlined in bold). Each sub-graph represents the temporal trend in NDVI of a specific African biome within the wintering area (colours along sub-graphs correspond to these used in the map). In each sub-graph, points represent average NDVI from November (year "i-1") to March (year "i"). Average NDVI values were obtained from bimonthly data developed by ECOCAST (available at <https://ecocast.arc.nasa.gov/data/pub/gimms/3g.v0/>). A land cover classification layer was used to clip each specific African biome (available at <http://neo.sci.gsfc.nasa.gov/view.php?datasetId=MCD12C1_T1>). The map was generated in ArcGis 10.2.2. (<http://support.esri.com/es/Products/Desktop/arcgis-desktop/arcmap/10-2-2>).

**
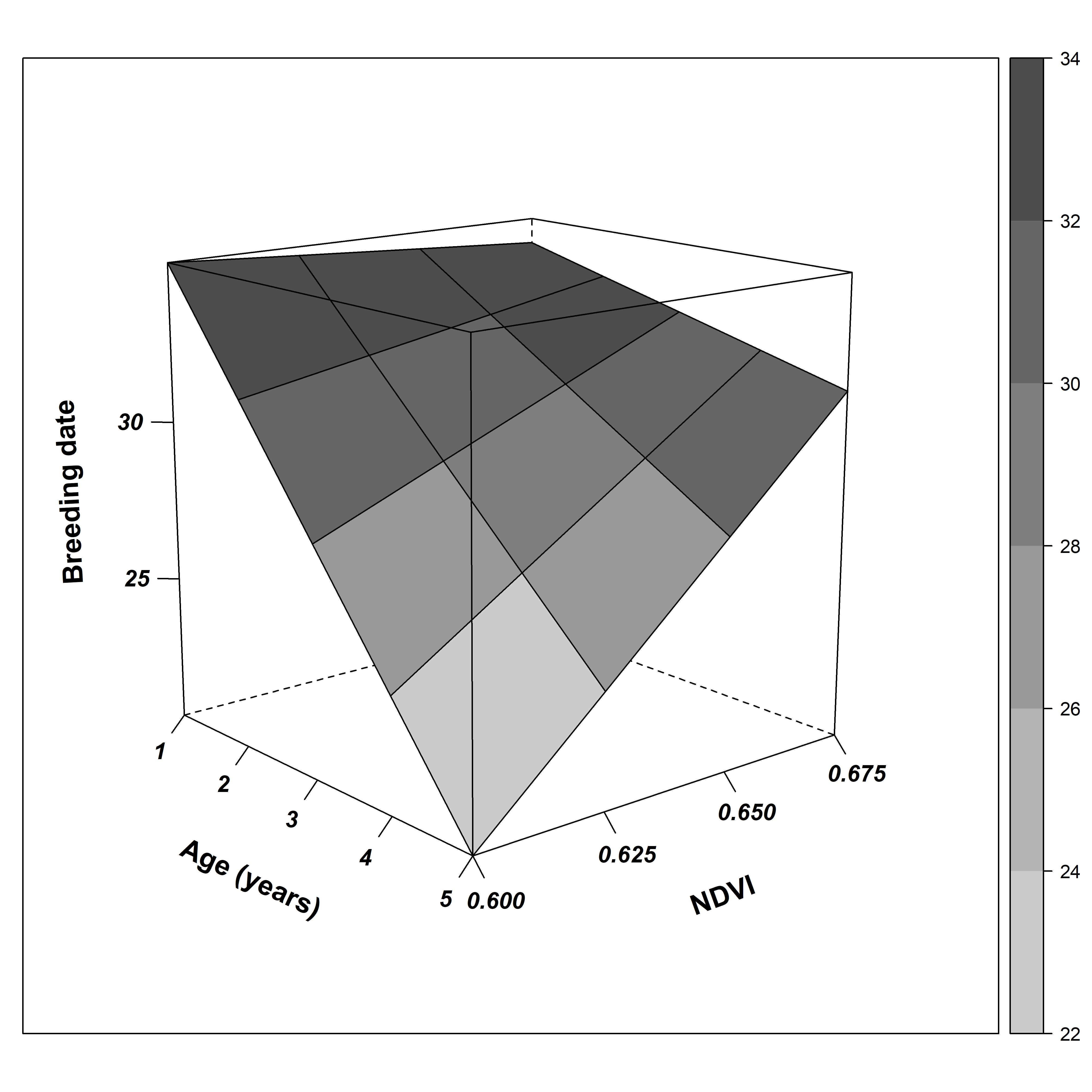
**

**Figure S2.** Three-dimensional surface plot showing the relationship between age, NDVI from winter areas and breeding date of males. Lines and grey scale represent predicted values obtained from the linear mixed model fitted for breeding date. A value of zero for breeding date corresponds to 1^st^ May.
