# Supplementary material for: Author Correction: Age-dependent carry-over effects in a long-distance migratory bird
Source: Sci Rep. 2021 Aug 11;11:16632. doi: 10.1038/s41598-021-94870-6 (PMC8357801; doi:10.1038/s41598-021-94870-6)
Supplement: Supplementary file 1 — Supplementary Information. [file 41598_2021_94870_MOESM1_ESM.docx]

**Supplementary Materials:**

**Age-dependent carry-over effects in a long distance migratory bird**

Cosme López Calderón^1^, Javier Balbontín Arenas^1^, Keith A. Hobson^2^ and Anders Pape Møller^3^

^1^*Department of Zoology, Faculty of Biology, Green Building, Avenue Reina Mercedes, E-41012 Seville, Spain*

^2^ *Department of Biology, University of Western Ontario, London, Ontario, Canada*

^3^ *Ecologie Systématique Evolution, Université Paris-Sud, CNRS, AgroParisTech, Université Paris-Saclay, F-91405 Orsay Cedex, France*

Correspondence to CLC:

Tel: (+34) 617 71 30 54

E-mail: clopez25@us.es

**Figure S1.** Temporal trends in NDVI across African biomes from our identified wintering areas. Pixels that were assigned to be the likely origin for >50% of our sample are determined by the grid (outlined in bold). Each sub-graph represents the temporal trend in NDVI of a specific African biome within the wintering area (colours along sub-graphs correspond to these used in the map). In each sub-graph, points represent average NDVI from November (year "i-1") to March (year "i"). Average NDVI values were obtained from bimonthly data developed by ECOCAST (available at <https://ecocast.arc.nasa.gov/data/pub/gimms/3g.v0/>). A land cover classification layer was used to clip each specific African biome (available at http://neo.sci.gsfc.nasa.gov/view.php?datasetId=MCD12C1_T1). The map was generated in ArcGis 10.2.2. (<http://support.esri.com/es/Products/Desktop/arcgis-desktop/arcmap/10-2-2>).

**Figure S2.** Three-dimensional surface plot showing the relationship between age, NDVI from winter areas and breeding date of females. Lines and grey scale represent predicted values obtained from the linear model fitted for breeding date (using the standardized path coefficient). A value of zero for breeding date corresponds to 1^st^ January.

**Figure S3.** Three-dimensional surface plot showing the relationship between age, NDVI from wintering areas and clutch size of females. Lines and grey scale represent predicted values obtained after calculating the indirect effect of the interaction between age and NDVI on clutch size. This indirect effect was calculated by multiplying standardized path coefficients connecting the interaction term with the number of eggs laid.
